# Performance Evaluation of Red Clay Binder with Epoxy Emulsion for Autonomous Rammed Earth Construction

**DOI:** 10.3390/polym12092050

**Published:** 2020-09-08

**Authors:** Jinsung Kim, Hyeonggil Choi, Keun-Byoung Yoon, Dong-Eun Lee

**Affiliations:** 1 School of Architecture, Civil, Environment and Energy Engineering, Kyungpook National University, Daegu 41566, Korea; kjs07406@knu.ac.kr; 2 Department of Polymer Science and Engineering, Kyungpook National University, Daegu 41566, Korea; kbyoon@knu.ac.kr

**Keywords:** autonomous rammed earth construction, soil stabilizer, red clay, epoxy emulsion, mechanical properties, microstructure

## Abstract

Existing rammed earth construction methods have disadvantages such as increased initial costs for manufacturing the large formwork and increased labor costs owing to the labor-intensive construction techniques involved. To address the limitations of the existing rammed earth construction methods, an autonomous rammed earth construction method was introduced herein. When constructing an autonomous rammed-earth construction method, an alternative means of assuring the performance at the initial age of the binder in terms of materials is needed. In this study, in order to satisfy the performance of the red clay binder, epoxy emulsion was added to analyze the compressive strength, water loosening, shrinkage, rate of mass change, and microstructure in the range of the initial age. As a result of the analysis, the applicability of the epoxy emulsion was confirmed as a new additive for application to an autonomous rammed-earth construction method.

## 1. Introduction

Earth buildings utilize soil as their primary material, allowing these buildings to reflect the characteristics of the soil itself. These buildings have excellent humidity control, deodorization rate, and energy efficiency, and are also eco-friendly owing to the influence of far-infrared radiation [1,2,3,4]. Earth buildings are classified according to the construction methods used; the most commonly used include rammed earth construction, poured-earth construction, and adobe (earthen brick) construction. In particular, in rammed earth construction, wood or iron is used to fabricate an integrated formwork into which soil is placed; this form is then rammed with a 7–10 kg rammer to form an integrated wall or floor. This is currently the most commonly used construction method in many regions [5,6,7]. Rammed earth construction mainly uses red clay as the primary material, and red clay consists of silt with a diameter of 0.002–0.005 mm in the classification according to the particle size distribution of the soil [8,9]. In terms of the physical property standards for rammed earth construction, ACP-EEC(African, Caribbean and Pacific-European Economic Community) requires a compressive strength of 2.4 MPa after 28 d of curing, while the New Mexico Adobe and Rammed earth Building Code recommends a strength of 200–300 psi (1.38–2.07 MPa) [8,10].

In existing rammed earth construction methods, a large integrated formwork is created and filled with soil, and then directly rammed with a vibrator to build the bearing wall [11,12,13,14]. This increases not only the initial costs to manufacture the formwork, but also the labor costs owing to the labor-intensive construction method. To address these problems, this study introduces a novel autonomous construction method that applies an autonomous design mechanism to existing rammed earth construction methods. Furthermore, the study assesses the addition of an epoxy emulsion to the red clay binder to enhance its performance.

**Figure 1:**
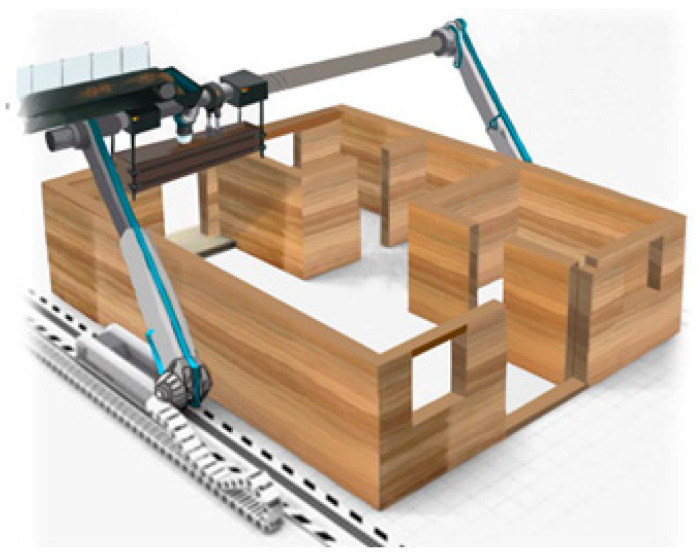
Schematic diagram of the autonomous rammed earth construction method.

Figure 1 shows a schematic diagram of the autonomous rammed earth construction method. Autonomous rammed earth construction involves a less labor force-based construction automation system that operates from the soil laying stage to mixing and compaction through an equipment automation process. The system performs mixing, transport, and compaction using an automatic mixer, extruder, auto-movable formwork, and three-axis automatic rammer, thus enabling high-quality construction. In addition, as autonomous rammed earth construction can be used to build modular movable formworks through an autonomous process, it enables faster construction speeds than existing rammed earth construction methods. Accordingly, in terms of the materials used in construction, the early-age strength development of the red clay binder is important. On the other hand, in terms of the material of the binder, in the case of the existing rammed earth construction method, lime or cement has been mainly used as an additive to improve the performance of the binder. However, in the case of lime, the exothermic temperature rises to 80 °C in the construction process through reaction with water, and there is a disadvantage in that caution is required during work due to the occurrence of considerable heat. In the case of cement, there are disadvantages in that the inherent characteristics of the soil itself deteriorate when more than an appropriate amount is added, as well as the environmental problems that arise in the cement production process. In the case of previous studies, about 8–10% of cement was suggested for reinforcing the strength of the soil [15,16]. In addition, when the autonomous rammed earth construction method is implemented, the strength of the bonding material at the initial age is important because of the improvement of the construction speed due to the application of the automated process. Therefore, to improve the early-age strength of the red clay binder when applying autonomous rammed earth construction, an epoxy emulsion was added to the aqueous polymer solution in this study; the applicability of this epoxy addition was confirmed in our preliminary research. In terms of the evaluation indices, the compressive strength, water loosening, shrinkage, rate of mass change, and microstructure were analyzed to establish the effectiveness of the epoxy emulsion.

## 2. Materials and Methods

### 2.1. Preliminary Experiment

Prior to the experiments in this study, a preliminary experiment was performed to select the optimal formulation of the epoxy emulsion, which is composed of the epoxy and a hardener. The preliminary research results demonstrated that satisfactory performance could not be obtained with use of the epoxy and hardener alone. Thus, the total amount of epoxy emulsion was selected by considering the viscosity of the epoxy and hardener. Four epoxy emulsion concentrations were used for the red clay + polymer aqueous solution + epoxy emulsion (RPE) test specimens: 4.0, 5.6, 6.8, and 8.0%. In terms of the evaluation indices, the compressive strength was measured at 3, 6, 12, and 24 h of aging.

**Figure 2:**
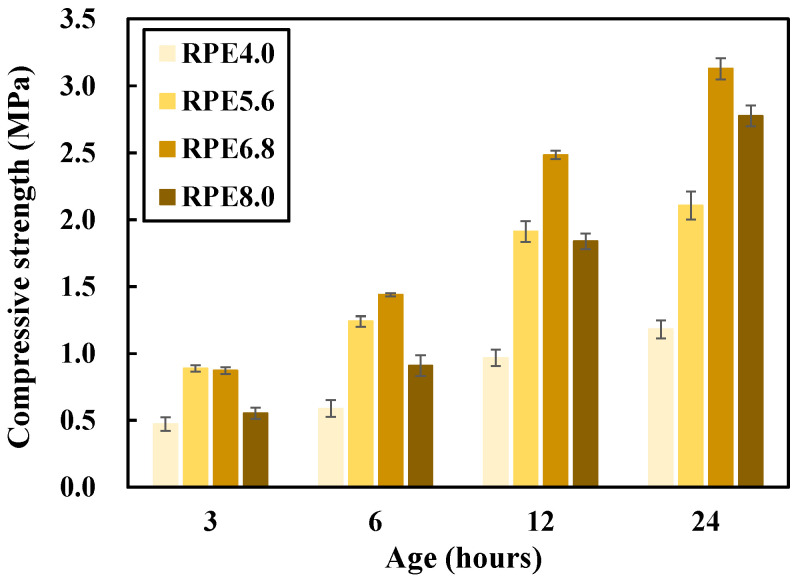
Compressive strength measurement results used to select the optimal concentration of epoxy emulsion in the red clay binder.

Figure 2 shows the compressive strength measurement results used to select the optimal concentration of epoxy emulsion. The RPE6.8 (6.8% mixing amount of epoxy emulsion) specimen exhibited higher compressive strengths than the other specimens. Moreover, a compressive strength of 2.4 MPa was achieved within just 12 h of aging, thus satisfying the requirement specified in rammed earth construction regulations at 28 d [8]. Therefore, this study selected RPE6.8 as the optimal concentration of epoxy emulsion to improve the performance (e.g., the strength and durability) of the red clay binder at the early age.

### 2.2. Main Experiment

#### 2.2.1. Materials

Table 1 lists the chemical compositions of the materials used in the experiments. The chemical composition analysis of red clay and ordinary Portland cement (OPC) was performed by X-ray fluorescence (XRF) method. Samples were prepared in powder form and then subjected to pretreatment and measured using a Wavelength Dispersive X-ray Fluorescence spectrometer (Bruker Korea, Seongnam, Republic of Korea). After the red clay was allowed to dry naturally, it was sieved using a 2 mm sieve to ensure a uniform particle size. For the cement, OPC of type KS L 5201 (Asia cement, Seoul, Republic of Korea) was used. The density of the cement was 3.12 g/cm³, and the fineness was 3500 cm^2^/g.

The aqueous polymer solution was in the form of poly(AA-co-AM), in which the monomer acrylic acid (AA) and acrylamide (AM) were copolymerized. The intrinsic viscosity of the polymer aqueous solution was 2–3 dL/g.

Table 2 and Table 3 list the physical and chemical properties of the epoxy and hardener used in these experiments, respectively [17]. The epoxy (KEM-101-50, KUKDO CHEMICAL, Seoul, Republic of Korea), which is a solid epoxy resin created in liquid form, is a general quick-drying epoxy with a water-based coating. The hardener (KH-700, KUKDO CHEMICAL, Seoul, Republic of Korea), which contains a polyamine, is a general hardener with excellent water and chemical resistance, high gloss, and a water-based coating.

#### 2.2.2. Experimental Plan

The experimental plan of this study is summarized in Table 4. A total of three formulation levels for the specimens were configured: RP (red clay + polymer aqueous solution), RPC (red clay + polymer aqueous solution + cement), and RPE (red clay + polymer aqueous solution + epoxy emulsion). During mixing, 5 wt% binder was added to the cement through internal replacement. For the polymer aqueous solution and epoxy emulsion, 8 wt% binder was added through external replacement considering the polymerization conditions and material viscosity. To ensure uniform consolidation, a universal testing machine was used to manufacture the specimen to a consolidation degree of 2 MPa. Curing was conducted at a constant temperature of 20 °C and relative humidity of 60%. In terms of the evaluation indices, the compressive strength and water loosening were measured to determine the curing properties. To investigate the deformation, the shrinkage and rate of mass change were measured. Microstructural analyses were performed using scanning electron microscopy (SEM, SEC, Suwon, Republic of Korea) and mercury intrusion porosimetry (MIP, Micromeritice, Norcross, GA, USA). Moreover, to investigate the curing properties of the red clay binder with varying temperature, the compressive strength under different curing conditions was measured.

#### 2.2.3. Experimental Methods

##### Specimen Production Process

A mortar mixer was used to mechanically mix the formulation with the red clay binder. First, the sample was weighed, poured into the mixer container, and dry mixed for 30 s. The additives were then added uniformly, and the sample was mixed for an additional 1 min. The binder that had become attached to the bottom and wall surfaces of the mixer container was then removed and collected in the center of the container, after which it was mixed again for 1 min. In the epoxy emulsion, to prevent agglomeration due to the viscosity of the epoxy and hardener, the epoxy and hardener were each weighed using a 20 mL laboratory syringe in a beaker in which the polymer aqueous solution had previously been weighed. The epoxy emulsion was then mixed for at least 1 min using a reagent spoon and added to a mortar mixer container.

To produce a specimen with a size of 50 mm × 50 mm × 50 mm, 240 g of the red clay binder was weighed. One-third of the weighed sample was then placed into each of three molds and consolidated to 2 MPa using a universal material tester to ensure uniform compaction. After reaching 2 MPa, the consolidation was maintained for 1 min to produce the specimen. The prepared specimens were cured at a constant temperature of 20 °C and relative humidity of 60%.

##### Compressive Strength

To measure the compressive strength based on specification KS L 5105 [18], three specimens of 50 mm × 50 mm × 50 mm were prepared according to the specified mixing conditions. The specimens were then measured with a hydraulic universal material tester at 3, 6, 12, and 24 h of aging.

Meanwhile, to investigate the effect of curing temperature on the compressive strength, the compressive strength of the RPE specimens was measured at an age of 24 h after curing at temperatures of 5, 20, and 35 °C.

##### Water Loosening

Among the specimens cured for 7 d, the specimens coated with inorganic ceramic resin (CERAST red clay waterproofing coating agent) and uncoated specimens were separated, and the water loosening behavior was observed. First, an inorganic ceramic resin was applied to the specimens with sizes of 50 mm × 50 mm × 50 mm, and a primary coating was applied to permeate the coating agent. After 30 min, the coating was reapplied to complete the coating of the specimen. Then, each specimen was immersed and observed at 3 h intervals for 48 h.

##### Shrinkage and Rate of Mass Change

Specimens with sizes of 50 mm × 50 mm × 50 mm were prepared and cured for 1 d at a constant temperature of 20 °C and relative humidity of 60%, after which the shrinkage was measured. First, a pressure sensitive (PS; Tokyo Measuring Instruments, Tokyo, Japan) adhesive was applied and reapplied to remove the surface voids of the specimen. Then, strain gauges (PFL-10-11-1LJC) were attached to both sides of the specimen with a cyanoacrylate (CN; Tokyo Measuring Instruments, Tokyo, Japan) adhesive. The strain gauges were then connected to a data logger (TDS-303) and the strain was measured at 10 min intervals until 28 d of aging.

Meanwhile, specimens with sizes of 50 mm × 50 mm × 50 mm were produced, the mass immediately after demolding was recorded, and the rate of mass change was measured at ages of 1, 3, 7, 14, 21, and 28 d at a constant temperature of 20 °C and relative humidity of 60%.

##### Scanning Electron Microscopy

Powder samples taken at 24 h of aging were coated with platinum, and the microstructure was observed at magnifications of ×3000 and ×5000 using a scanning electron microscope (SNE-3200M) at an acceleration voltage of 15 kV.

##### Mercury Intrusion Porosimetry

Powder samples collected at 24 h of aging were completely dried in a drying furnace at a temperature of 60 °C. Non-wetting mercury was then pressurized at 0–60,000 psi, and the total cumulative porosity and pore diameter of the samples were measured based on the amount of intrusion using a porosimeter (AutoPore IV 9520).

## 3. Results and Discussion

### 3.1. Compressive Strength

**Figure 3:**
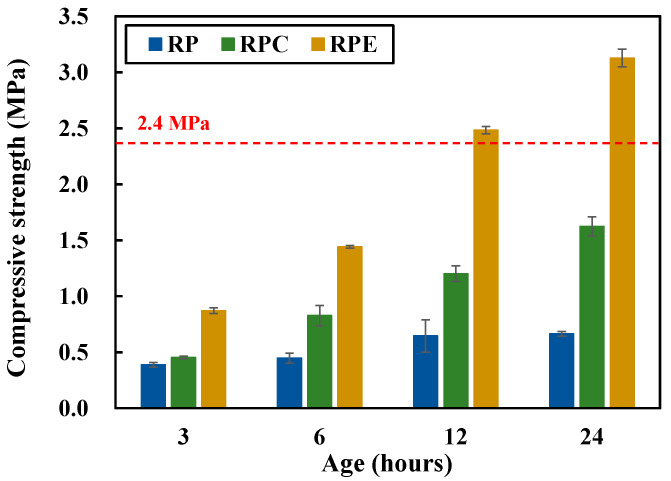
Compressive strength results for red clay binder specimens.

Figure 3 shows the compressive strength results for the red clay binder specimens. The compressive strength was measured at 3, 6, 12, and 24 h of aging. The compressive strength of the RPE (red clay + polymer aqueous solution + epoxy emulsion) specimens was substantially higher compared with that of the RP (red clay + polymer aqueous solution) and RPC (red clay + polymer aqueous solution + cement) specimens. This is attributed to the curing mechanisms of the epoxy and hardener in the epoxy emulsion. In the epoxy curing mechanism, the molecular structure of the epoxy group (C–O–C) has a triangular shape of primary bound amines. This then binds with a hydroxy group (–OH) to form the secondary amine form, which is a polymer chain. Owing to the increase in binding reactions, the size of the molecular bonds increases, and the viscosity increases as the epoxy cures [19,20,21,22]. Meanwhile, the hardener breaks the bond of the epoxy group (C–O–C) molecular structure, which is the primary amine form of the epoxy, resulting in the formation of two chains and an increase in the effectiveness of the epoxy [23,24].

The RPE specimen reached a compressive strength of 2.4 MPa within just 12 h of aging, which satisfies the requirement for earthen walls specified in the ACP-EEC rammed earth construction regulations at 28 d of curing [8]. This suggests that the epoxy emulsion can be applied as an additive for strength development at an early age, which is a requirement for materials used for autonomous rammed earth construction.

### 3.2. Water Loosening

Table 5 summarizes the water loosening results with respect to the addition of epoxy emulsion. The specimens with and without coating agents were separated and their water loosening characteristics were observed. In the red clay binder without a coating agent, the bonds between the red clay particles began to loosen immediately after immersion at all formulation levels. Water loosening then proceeded rapidly for the first 3 h of immersion and was completed after 24 h of immersion.

In contrast, the coated red clay binders achieved water resistance at all formulation levels based on the water loosening results obtained up to 48 h after immersion. This indicates that when applying red clay binders in the field, a waterproof coating agent must be applied to ensure water resistance.

### 3.3. Shrinkage and Rate of Mass Change

**Figure 4:**
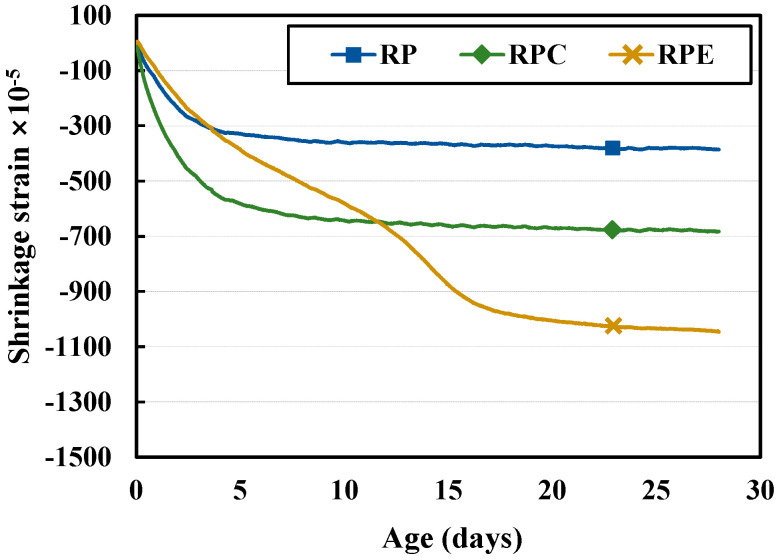
Shrinkage results for the red clay binder specimens.

**Figure 5:**
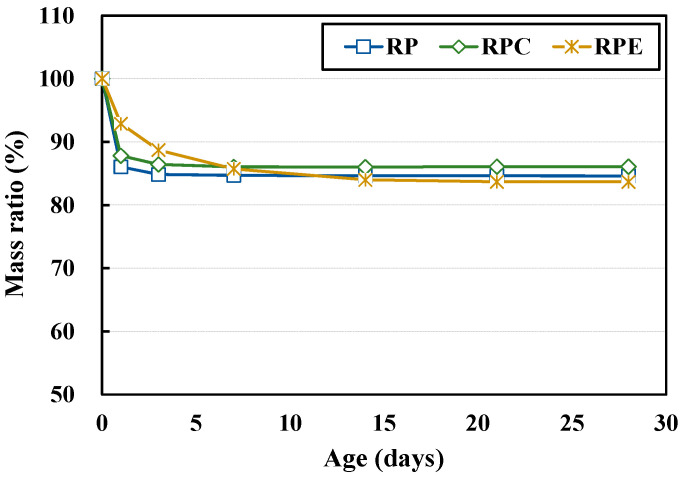
Rates of mass change in the red clay binder specimens.

Figure 4 and Figure 5 show the results for the shrinkage and rate of mass change, respectively, of the red clay binder specimens. In the RP and RPC specimens, which did not contain epoxy emulsion, shrinkage occurred at an early age, and the slope of the strain curve became gradual after 5 d of aging. In the RP specimens, as the solid polymer content of the aqueous poly(AA-co-AM) polymer solution formed hydrogen bonds with the silica particles in the red clay, the moisture gradually evaporated, thereby increasing shrinkage. In addition, in the RPC specimens, the shrinkage caused by the solid polymer content and added cement, introduces moisture from the red clay binder to participate in hydration reactions, which causes the volumetric deformation of the red clay binder and increases shrinkage. On the other hand, in the case of the RPE test specimen, the shrinkage was continuously increased, and after 5 d, the shrinkage deformation was greater than that of the RP test specimen, and after 12 d, the contraction deformation was larger than that of the RPC specimen. This is attributed to the rapid setting of the red clay binder due to the molecular structure of the epoxy and hardener in the epoxy emulsion, which increased the attractive force between the red clay particles with age and thus increased shrinkage. This suggests a need to investigate the curing properties and shrinkage of RPE further at longer aging times.

Though the rates of mass change did not differ considerably overall, the RPE specimens had a smaller rate of mass change than the RP and RPC specimens at early ages. This is attributed to the increased attractive force between the red clay particles due to the curing mechanisms of the epoxy and hardener in the epoxy emulsion. Specifically, owing to the rapid setting of the red clay binder caused by the molecular bonds of the epoxy and hardener, the rate of mass change due to drying is small but the shrinkage is large.

### 3.4. Scanning Electron Microscopy (SEM)

**Figure 6:**
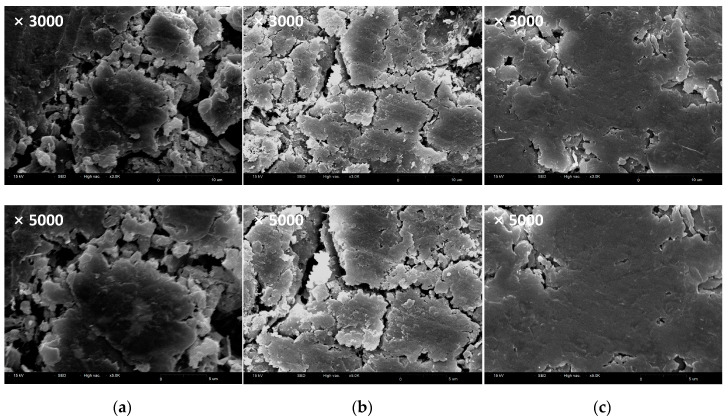
SEM micrographs of (**a**) RP, (**b**) RPC, and (**c**) RPE specimens at 24 h (scale bar: ×3000, 10 µm; ×5000, 5 µm).

Figure 6 shows SEM micrographs of the red clay binder specimens at 24 h of aging. The RP and RPC specimens (Figure 6a,b), which did not contain the epoxy emulsion, showed partially smooth surfaces. This is likely to be because the solid polymer content in the aqueous polymer solution effectively formed hydrogen bonds with the silica particles in the red clay as secondary bonds, causing the red clay binder to agglomerate.

On the other hand, the RPE specimen (Figure 6c), which contained epoxy emulsion, exhibited a wider and smoother plate-shaped surface than the other two specimen types at the same magnification. This is attributed to an increase in the effect of uniform red clay particle agglomeration, which is caused by the increase in molecular bonding of secondary amines through bonding with the hydroxyl group (–OH) in the primary amines of the epoxy, as well as the curing mechanism of the hardener that promotes this reaction. Notably, as the RPE sample achieved curing in a shorter time than the other two formulations, a wider and smoother plate shape overall was confirmed.

### 3.5. Mercury Intrusion Porosimetry (MIP)

**Figure 7:**
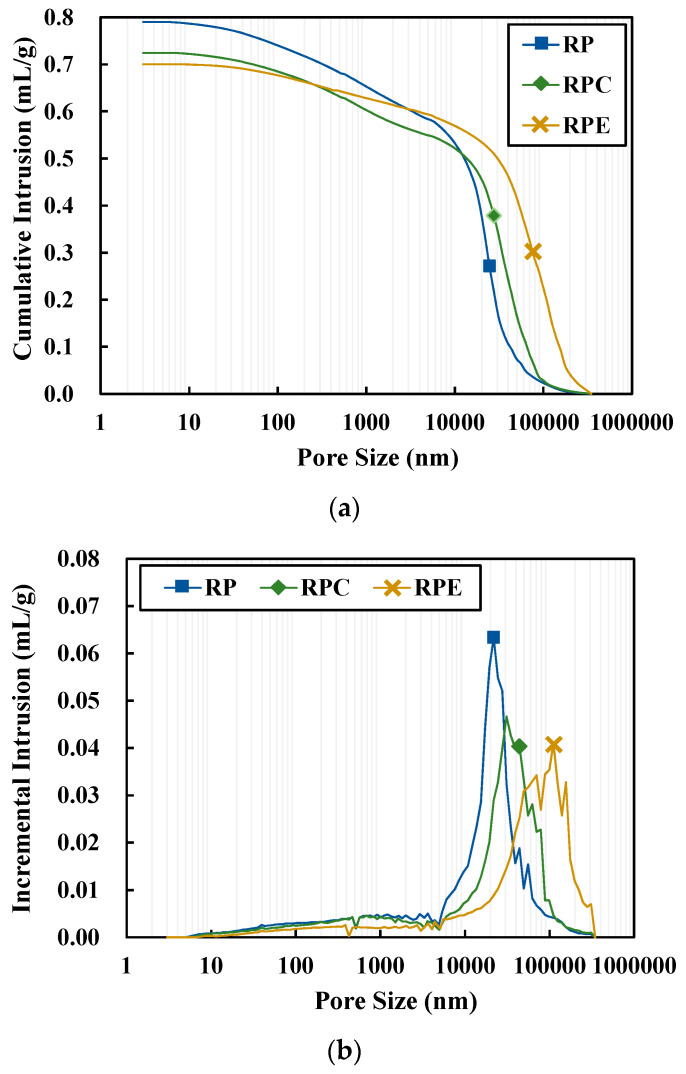
MIP results for the red clay binder specimens at 24 h. (**a**) Cumulative intrusion and (**b**) incremental intrusion.

Figure 7 shows the MIP results for the red clay binder specimens at 24 h of aging, where Figure 7a,b depicts cumulative and incremental intrusion, respectively. The RPE specimens, which contained the epoxy emulsion, exhibited larger pore diameters than the other two formulations, although the total cumulative pore volume was smaller. In this regard, as noted in the SEM results, adding the epoxy emulsion caused a wider and smoother plate shape to form compared to the formulations without the epoxy emulsion. Although the total cumulative pore volume between the plate-shaped particles created as the red clay particles agglomerated is small, the gaps between the plate-shaped particles due to the wider plate shape increased, thus resulting in larger pore diameters in the RPE specimen than in the other two formulations.

### 3.6. Compressive Strength with Varying Curing Conditions

**Figure 8:**
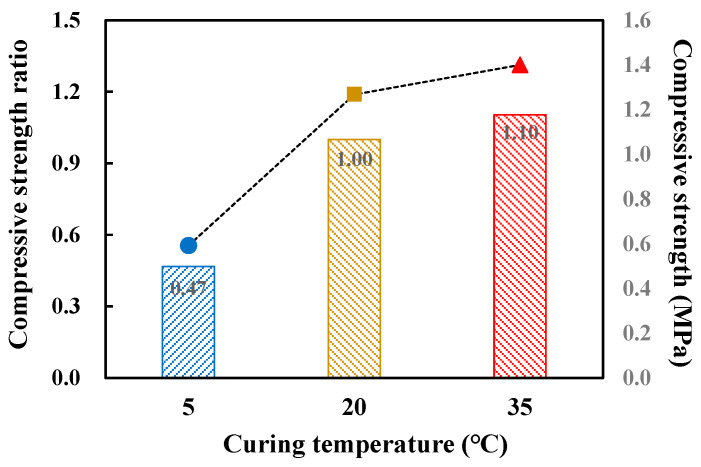
Compressive strength measurement results according to curing condition of red clay binder specimens at 24 h.

For the RPE formulation, which contains epoxy emulsion, to examine the curing properties of the red clay binder with varying temperature, the compressive strength was measured under different curing conditions. Figure 8 shows the compressive strength measurement results for the red clay binder specimens at 24 h of aging under different curing conditions. The compressive strength measured after curing the prepared specimens at 20 °C was set as 1, and the relative strength ratios of the compressive strengths measured at 5 and 35 °C were calculated. The strength development at a curing temperature of 35 °C was similar to that at 20 °C. In contrast, the compressive strength at 5 °C was approximately 50% of that at the other two curing temperatures. This suggests that for construction in winter conditions at below average temperatures, an insulated curing process is necessary.

## 4. Conclusions

This study evaluated the performance of a red clay binder with the addition of an epoxy emulsion by analyzing the compressive strength, water loosening, shrinkage, and microstructure of the red clay binder. The compressive strength characteristics were also evaluated under different curing conditions. In these evaluations, the formulation condition of the epoxy emulsion was varied. The following conclusions were obtained in this study.

1. Adding an epoxy emulsion increased the attractive force between the red clay binder particles owing to the hardening mechanism of the epoxy and hardener in the epoxy emulsion, thereby causing the red clay particles to agglomerate in a wide plate shape and thus improving the strength of the red clay binder.
2. In general, the use of cement as a solidifying material has been suggested to develop the strength of the binder in the rammed earth construction method [25,26,27]. On the other hand, the epoxy emulsion-added test specimen (RPE) showed higher strength development in the range of early age compared to the cement-added test specimen (RPC) due to the curing mechanism of each of the epoxy and the hardener.
3. The red clay binder without a coating agent could not achieve water resistance. On the other hand, applying a coating agent enabled the specimen to achieve water resistance, indicating that when applying red clay binders in the field, a coating agent must be applied to ensure water resistance.
4. Owing to the molecular bonding between the epoxy and hardener, the red clay binder set rapidly. Accordingly, it was determined that shrinkage increases as the attractive force between the red clay particles increases with age. In addition, the red clay particles agglomerated in a wide plate shape and the pore diameter due to the gaps between the particles increased, while the total cumulative pore volume decreased.
5. The epoxy emulsion applied in this study enhanced the strength and durability of the red clay binder as a result of the curing mechanisms of the epoxy and hardener. This suggests that an epoxy emulsion can be applied to satisfy the performance requirements for autonomous rammed earth construction, such as improving the durability and strength at an early age.

## Figures and Tables

**Table 1:** Chemical Compositions of Materials Used in the Experiments.

Materials	Chemical Composition (%)										
	SiO_2_	Al_2_O_3_	K_2_O	Fe_2_O_3_	TiO_2_	MgO	CaO	Pd	Ru	ZrO_2_	LOI
Red clay	56.79	24.87	4.63	3.99	0.74	0.62	0.13	0.07	0.06	0.04	7.95
OPC	20.70	6.20	0.84	3.10	-	2.80	62.20	-	-	-	1.96

Note: LOI: Loss On Ignition.

**Table 2:** Physical and Chemical Properties of the Epoxy Used in the Experiments.

Spec	EEW (g/eq)	Viscosity (cps@25 °C)	Non-Volatile Content (wt%)
KEM-101-50	450–550	1000–10000	47

Note: EEW is the epoxy equivalent weight.

**Table 3:** Physical and Chemical Properties of the Hardener Used in the Experiments.

Spec	TAV (mgKOH/g)	Viscosity (cps@25 °C)	AHEW (g/eq)	Non-Volatile Content (wt%)
KH-700	190–250	3000–10000	170	80

Note: TAV is the total amine value, AHEW is the amine hydrogen equivalent weight.

**Table 4:** Experimental Plan.

Type	Binder (wt%)		PA (B × wt%)	EM (E + H) (B × wt%)	Consolidation Condition	Curing Condition	Evaluation Items
	R	C					
RP	100	-	8	-	2 MPa	20 °C RH 60%	Compressive strength Water loosening Shrinkage Rate of mass change Scanning electron microscopy Mercury intrusion porosimetry Compressive strength with curing condition
RPC	95	5	8				
RPE	100	-	8	6.8 (E:H = 11:6)			

Note: R: red clay, C: cement, PA: polymer aqueous solution, EM: epoxy emulsion, E: epoxy, H: hardener. Note: P (polymer) 5% = PA × wt%.

**Table 5:** Water Loosening Results for Red Clay Binder Specimens.

Without Coating					With Coating				
Spec.	Flooding Times (h)				Spec.	Flooding Times (h)			
	0	3	24	48		0	3	24	48
RP	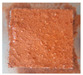	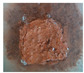	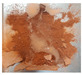	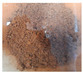	RP	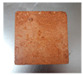	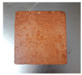	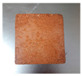	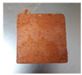
RPC	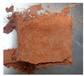	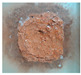	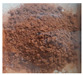	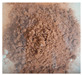	RPC	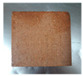	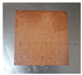	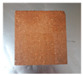	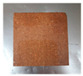
RPE	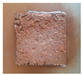	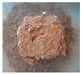	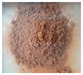	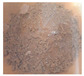	RPE	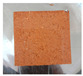	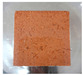	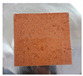	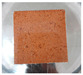
